# Cardiomyocyte-Restricted Deletion of PPAR**β**/**δ** in PPAR**α**-Null Mice Causes Impaired Mitochondrial Biogenesis and Defense, but No Further Depression of Myocardial Fatty Acid Oxidation

**DOI:** 10.1155/2011/372854

**Published:** 2011-09-05

**Authors:** Jian Liu, Peiyong Wang, Lan He, Yuquan Li, Jinwen Luo, Lihong Cheng, Qianhong Qin, Lawrence A. Brako, Woo-kuen Lo, William Lewis, Qinglin Yang

**Affiliations:** ^1^Department of Nutrition Sciences, University of Alabama at Birmingham, 1675 University Boulevard, Birmingham, AL 35294-3360, USA; ^2^Department of Anatomy, Second Military Medical University, Shanghai 200433, China; ^3^Department of Cardio Thoracic Surgery, Hunan Children's Hospital, Changsha 410007, China; ^4^Division of Cardiology, Department of Medicine, Emory University, Atlanta, GA 30322, USA; ^5^Department of Anatomy, Morehouse School of Medicine, Atlanta, GA 30310, USA; ^6^Department of Pathology, Emory University, Atlanta, GA 30322, USA

## Abstract

It is well documented that PPAR**α** and PPAR**β**/**δ** share overlapping functions in regulating myocardial lipid metabolism. However, previous studies demonstrated that cardiomyocyte-restricted PPAR**β**/**δ** deficiency in mice leads to severe cardiac pathological development, whereas global PPAR**α** knockout shows a benign cardiac phenotype. It is unknown whether a PPAR**α**-null background would alter the pathological development in mice with cardiomyocyte-restricted PPAR**β**/**δ** deficiency. In the present study, a mouse model with long-term PPAR**β**/**δ** deficiency in PPAR**α**-null background showed a comparably reduced cardiac expression of lipid metabolism to those of single PPAR-deficient mouse models. The PPAR**α**-null background did not rescue or aggravate the cardiac pathological development linked to cardiomyocyte-restricted PPAR**β**/**δ** deficiency. Moreover, PPAR**α**-null did not alter the phenotypic development in adult mice with the short-term deletion of PPAR**β**/**δ** in their hearts, which showed mitochondrial abnormalities, depressed cardiac performance, and cardiac hypertrophy with attenuated expression of key factors in mitochondrial biogenesis and defense. The present study demonstrates that cardiomyocyte-restricted deletion of PPAR**β**/**δ** in PPAR**α**-null mice causes impaired mitochondrial biogenesis and defense, but no further depression of fatty acid oxidation. Therefore, PPAR**β**/**δ** is essential for maintaining mitochondrial biogenesis and defense in cardiomyocytes independent of PPAR**α**.

## 1. Introduction

Peroxisome proliferator-activated receptors (PPAR*α*, *β*/*δ*, and *γ*) form a family of ligand-dependent nuclear receptor transcription factors. It is well-documented that PPAR*α* and PPAR*β*/*δ* are important in transcriptional regulation of cardiac lipid metabolism [1, 2]. 

PPAR*α* is abundantly expressed in tissues with an elevated capacity for fatty acid oxidation (FAO), such as brown fat, liver, heart, and kidney [3]. The important role of PPAR*α* in controlling cardiac energy metabolism and function has been established by studies using both loss- and gain-of-function approaches in animal models [4, 5]. PPAR*α* regulates fatty acid homeostasis via transcriptional activation of genes encoding key enzymes in fatty acid metabolism. Despite diminished expression of FAO genes and a subsequently depressed FAO rate in the heart under basal conditions [6], PPAR*α*-null mice are viable with no overt pathological development, but no longer responsive to fasting or diabetes to upregulate FAO gene expression [7]. Interestingly, lipotoxic cardiomyopathy in mice with transgenic PPAR*γ* overexpression in the heart can be corrected in the PPAR*α*-null background [8]. Transgenic PPAR*α* overexpression in the heart also leads to lipid overload and cardiomyopathy [9]. PPAR*β*/*δ* is expressed ubiquitously throughout the body and at relatively high levels in cardiomyocytes [10, 11]. PPAR*β*/*δ* activates expression of genes that are involved in FAO independent of PPAR*α* in cardiomyocytes [10, 12, 13]. Our previous studies indicate that an early, long-term, cardiomyocyte-restricted- (CR-) PPAR*β*/*δ* deletion in mice impairs myocardial FAO and bioenergetics, leading to cardiac dysfunction, progressive myocardial lipid accumulation, cardiac hypertrophy, and congestive heart failure [12, 14]. Importantly, we recently discovered that PPAR*β*/*δ* is essential for the adult heart to maintain mitochondrial biogenesis and antioxidant defense [15, 16, 22].

It becomes clear that both PPAR*α* and PPAR*β*/*δ* regulate an overlapping set of genes involved in myocardial FAO [2, 17], albeit cardiac expression of PPAR*α* is more responsive than PPAR*β*/*δ* to dietary stress [18]. It remains to be determined whether the absence of PPAR*α* would predispose the pathological development induced by PPAR*β*/*δ* deficiency in the heart due to their overlapping and specific functions. While it is clear that PPAR*β*/*δ* is important in cardiac mitochondrial function under basal conditions, PPAR*α* has also been shown to be involved in the regulation of mitochondrial function in the heart under certain conditions (e.g., during the prediabetic, insulin-resistant stage) [19]. However, it is unknown whether a PPAR*α*-null background would alter the pathological development in mice with cardiomyocyte-restricted PPAR*β*/*δ* deficiency. The present study tested the hypothesis that PPAR*α* is required for the detrimental effects of PPAR*β*/*δ* deficiency in the heart on myocardial FAO and mitochondrial biology by studying a mouse model of cardiomyocyte-restricted PPAR*β*/*δ* deficiency with the absence of systemic PPAR*α*.

## 2. Methods

### 2.1. Mouse Models of PPAR*α* and PPAR*β*/*δ* Knockout

Long-term cardiomyocyte-restricted PPAR*δ* knockout mice (CR-PPAR*β*/*δ* ^−/−^) [12, 15] were obtained by mating the PPAR*β*/*δ* ^flox/flox^ line [20] with the *α*-MyHC-Cre line [21]. The tamoxifen inducible cardiomyocytes-restricted PPAR*β*/*δ* knockout model (TMPD) has been described [22]. It was obtained by mating the PPAR*β*/*δ* ^flox/flox^ line with the *α*-MyHC-driven Mer-Cre-Mer (MCM) overexpression [23]. The above resulted lines were mated with PPAR*α*-null mice [24] to obtain cardiac double knockout lines (CR-Pd/Pa and TMPDPA, resp.). All mice were in pure C57/B6 background. 

Cardiomyocyte-restricted PPAR*β*/*δ* knockout was induced in 10 week-old mice by 5 days of Tamoxifen treatment (2 mg/200 *μ*L in sunflower oil, intraperitoneal injection). The short-term effect of PPAR*β*/*δ* deficiency was assessed in mice two weeks after the 5-day injection of tamoxifen. There was no body weight difference in mice from all of the experimental groups. All experimental procedures were conducted in accordance with the Guide for Care and Use of Laboratory Animals of the National Institutes of Health and were approved by the Institutional Animal Care and Use Committee of the University of Alabama at Birmingham (UAB).

### 2.2. Transcript Analyses

Total RNA samples were extracted from left ventricles using an RNA extraction kit (Qiagen) according to the manufacturer's instructions. Quantitative real-time RT-PCR analyses were carried out using the Roche LightCycler 480 system (Roche) to determine transcript levels of target genes. Real-time PCR results from each gene/primer pair were normalized to results of *β*-actin and compared across conditions. 

### 2.3. Protein Analysis

Cytoplasmic or nuclear protein samples were extracted from left ventricles with NE-PER nuclear and cytoplasmic protein extraction reagents (Thermo Pierce 78833). Western blots were conducted using commercially available antibodies. The immunoblotting images were captured using KODAK Image Station 4000R (Carestream Health Inc.) by developing the membranes in SuperSignal West substrates (Thermo Scientific, 34080 or 34076) and analyzed with KODAK IM software (Ver 4.5.1). All antibodies were purchased from commercial sources: PPAR*β*/*δ*, FABP, CPT1b, and Catalase (Abcam); Glut1, Glut4, PGC-1*α*, Cyt b, DRP1, Fis1, SOD2, and GAPDH (Santa Cruz Biotechnology); TFam (Aviva); Cyt C (Invitrogen); Mfn2, and pan-actin (Sigma Aldrich); SOD1 (Biodesign International).

### 2.4. Analysis of Mitochondrial DNA Copies

The mitochondrial DNA copy number was evaluated by adapting to methods described previously [25]. Total genomic DNA was isolated from left ventricles, processed by standard procedures using a DNA extracting kit (Qiagen), digested with NcoI, and subjected to real-time qPCR analysis. Cytochrome b (cytb) was employed as a mitochondrial DNA (mtDNA) marker and the regulator of calcineurin 1 (rcan1) as a nuclear DNA (nDNA) marker to quantify the amount of mtDNA. 

### 2.5. Analyses of Myocardial TAG

Myocardial triglyceride contents in adult mice were assayed using lipid diagnostic kits (Wako Chemicals USA, Inc.). Fresh samples were used and harvested from mice subjected to fed (35-week old) or 12-hour fasting conditions (50-week old). For myocardial lipid extraction, left ventricular tissues were homogenized with ice-cold chloroform-methanol-water mixture (2 : 1 : 0.8) for 2 min. Additional chloroform and water were added to separate the organic and aqueous layers. After centrifugation, the aqueous layer was removed, and the chloroform layer was decanted and evaporated at 70°C. The residue was dissolved in 0.5 mL of isopropanol. 

### 2.6. Transmission Electron Microscopy (TEM)

To obtain tissue for TEM, hearts of anesthetized mice were perfused under gravity with 3.5% glutaraldehyde in cardioplegic solution (25 mmol/L KCl, 5% dextrose in PBS, pH 7.4) for two minutes followed by perfusion with 3.5% glutaraldehyde in a 0.1 mol/L cacodylate buffer, pH 7.3 for another 2 minutes. We take a section (1 mm cubed) from the apex of the left ventricle. Sections were assessed by toluidine blue staining, and the longitudinal sections were examined by TEM.

### 2.7. [9, 10-^3^H] Palmitate Oxidation and [^14^C] Glucose Oxidation Assay in Isolated Working Heart

We measured the rate of palmitate and glucose oxidation using an isolated working heart preparation as described previously [16, 26]. The mouse heart was perfused with Krebs-Henseleit solution containing 3% BSA (essentially FA, Intergen Corporation), 5 mmol/L glucose, 100 *μ*U/mL insulin, and 0.4 mmol/L palmitate. Hearts were perfused at a constant left atrial preload pressure of 7 mmHg and a constant aortic afterload pressure of 50 mmHg. Myocardial FA and glucose oxidation rates were determined by quantitative collection of ^3^H_2_O or ^14^CO_2_ produced by hearts perfused with buffer containing [9,10-^ 3^H] palmitate (0.1 *μ*Ci/mL, MP Biomedicals) and [U-^ 14^C] glucose (0.1 *μ*Ci/mL, MP Biomedicals) for palmitate oxidation and glucose oxidation, respectively. ^3^H_2_O that was produced during palmitate oxidation was separated with a water vapor exchange method [27]. Palmitate oxidation rates were calculated from ^3^H_2_O production, taking into account the vapor exchange efficiency. ^14^CO_2_ produced during glucose oxidation was collected using a CO_2_ trapping method [28].

### 2.8. Mitochondrial Oxidative Stress

Oxidative stress of cardiac mitochondria was accessed using freshly isolated mitochondria, which were prepared with mitochondria isolation kits (SIGMA, MITOISO1) according to the protocols provided. Mitochondria potential was determined using the JC-1 assay kit (SIGMA) according to the manufacturer's instruction. Five *μ*g of mitochondria protein in 75 *μ*L of the JC-1 assay buffer was used. The relative fluorescence of the sample was measured using a time-drive method in a spectrofluorescent multiwell plate reader (Synergy HT, Bio-Tek) with 485/20 nm (excitation), 590/35 nm (emission). Aconitase activity was measured spectrophotometrically based on the instruction of the manufacturer (Sigma) with modification for a 96- well plate assay. The activity was calculated using *V*_max_.

### 2.9. Echocardiography Measurement

As described previously [16, 26], a high-resolution echocardiograph system (Visualsonics VEVO 770 System) was used to assess cardiac structure/function *in vivo* with a 35 MHz probe at various time points and before terminal experiments. Mice were anaesthetized by isoflurane inhalation. Heart rate was maintained at ~450 beats per minute, and the body temperature was maintained at 37°C by placing mice on a heating pad. We obtained IVS (intraventricular septum thickness), LVID (left ventricular internal dimension), LV volume, LVPW (left ventricular posterior wall thickness), EF%, and FS% under long-axis M-mode. All data and images were saved and analyzed by an Advanced Cardiovascular Package Software (Visualsonics VEVO 770 System).

### 2.10. Statistics Analysis

Data for comparison of two groups were analyzed using Student's *t*-test; otherwise, the data were analyzed by one factor or mixed, two-factor analysis of Variance (ANOVA) using GraphPad Prism software (GraphPad Software Inc.). Values of quantitative results were expressed as mean ± SEM. Differences between groups and treatments were regarded as significant at the *P* < 0.05 probability level.

## 3. Results

### 3.1. Early and Long-Term Cardiac PPAR*β*/*δ* Deficiency in PPAR*α*-Null Mice Leads to Phenotypic Changes Consistent with CR-PPAR*β*/*δ* ^−/−^ Mice

**Figure 1:**
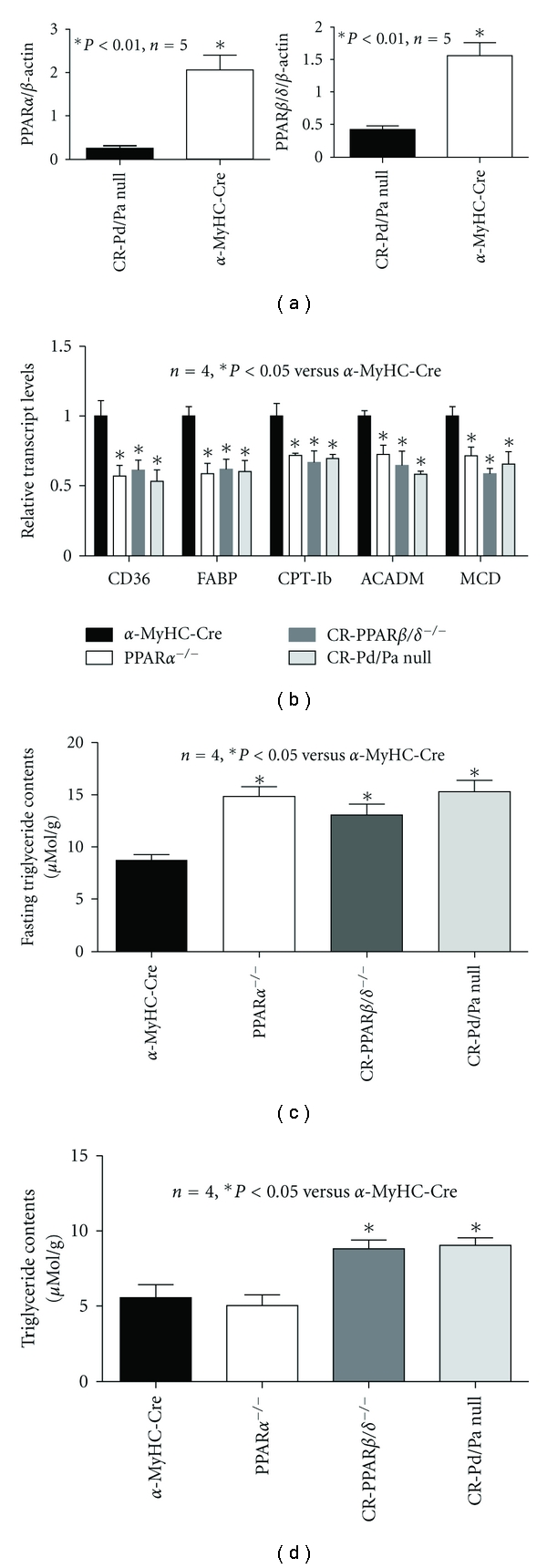
Transcript levels measured by real-time RT-PCR analyses. (a) Transcript expression levels of PPAR*α* and PPAR*β*/*δ* in CR-Pd/Pa-null and *α*-MyHC-Cre hearts. RNA samples were extracted from hearts of 35-week-old mice. (b) Transcript expression levels of CD36, FABP, CPT-Ib, ACADM, and MCD in *α*-MyHC-Cre, CR-PPAR*β*/*δ* ^−/−^, PPAR*α* ^−/−^, and CR-Pd/Pa-null hearts. RNA samples were extracted from hearts of ~35-week-old mice. (c) Myocardial triglyceride content was measured in mice subjected to fasting. (d) Myocardial triglyceride content was measured in mice without fasting.

**Figure 2:**
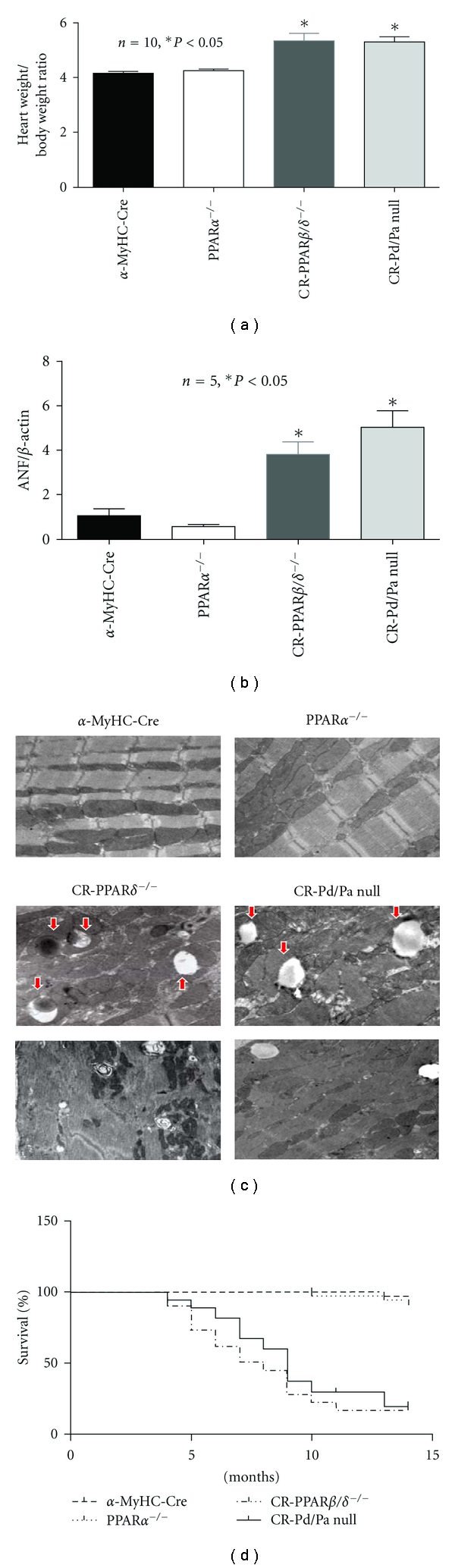
Cardiac pathology and survival rate. (a) Cardiac hypertrophy was estimated by heart weight/body weight ratio. (b) Transcript expression levels of ANF in *α*-MyHC-Cre and CR-Pd/Pa-null hearts. (c) Assessment of cardiac ultrastructure by Transmission Electron Microscopy (TEM): representative images are shown of heart sections from *α*-MyHC-Cre, PPAR*α* ^−/−^, CR-PPAR*β*/*δ* ^−/−^, and CR-Pd/Pa-null mice at the age of 35 weeks (image magnification: 12,000x). Arrows indicate lipid droplet. (d) Kaplan-Meier survival curves: survival rates of mice from *α*-MyHC-Cre, CR-PPAR*β*/*δ* ^−/−^, CR-Pd/Pa-null, and control groups after 15 months were analyzed by the log-rank test (CR-PPAR*β*/*δ* ^−/−^ or CR-Pd/Pa versus *α*-MyHC-Cre, *P* < 0.001).

We crossed the cardiomyocyte-restricted PPAR*β*/*δ* line (CR-PPAR*β*/*δ* ^−/−^) with the PPAR*α*-null line (PPAR*α* ^−/−^) to generate a mouse line with early and long-term cardiac PPAR*β*/*δ* deficiency in PPAR*α*-null background (CR-Pd/Pa-null) mice. Real-time PCR confirmed that PPAR*α* and PPAR*β*/*δ* in the CR-Pd/Pa-null heart were knockout or knockdown (Figure 1(a)). The CR-Pd/Pa-null hearts exhibited the attenuation of FAO genes, such as CD36, heart type fatty acid binding protein (FABP), carnitine palmitoyl transferase-Ib (CPT-Ib), and acyl-Coenzyme A dehydrogenase medium chain (ACADM), which was similar to either PPAR*α*-null or CR-PPAR*β*/*δ* ^−/−^ hearts (Figure 1(b)). Myocardial triglyceride contents were comparably elevated in fasting PPAR*α*-null, CR-PPAR*β*/*δ* ^−/−^, and CR-Pd/Pa-null mice (Figure 1(c)) and elevated only in fed CR-PPAR*β*/*δ* ^−/−^ and CR-Pd/Pa-null mice (Figure 1(d)). As with CR-PPAR*β*/*δ* ^−/−^ mice, CR-Pd/Pa-null mice exhibited cardiac hypertrophy, with an increased heart weight/body weight ratio and ventricular expression of the atrial natriuretic factor (ANF) after ~35 weeks of age (Figures 2(a) and 2(b)). Ultrastructural changes in CR-Pd/Pa-null hearts were identical to those in CR-PPAR*β*/*δ* ^−/−^ hearts, which displayed substantial mitochondrial shrinkage and depletion, increased number and sizes of lipid droplets and sarcomeric disruption (Figure 2(c)). Mitochondrial volume and mitochondrial DNA copy number were decreased in the CR-PPAR*β*/*δ* ^−/−^ and CR-Pd/Pa-null mice (Figures 1 a and b in Supplementary Material available at doi: 10.1155/2011/372854). Similar to CR-PPAR*β*/*δ* ^−/−^, most of the CR-Pd/Pa-null mice died from heart failure by 13 months of age (Figure 2(d)). These results demonstrate the essential roles of PPAR*β*/*δ* in cardiac structure/function for all developmental stages through adulthood. 

### 3.2. Short-Term Cardiac PPAR*β*/*δ* Deficiency in PPAR*α*-Null Mice Does Not Further Suppress Myocardial Lipid Metabolism

**Figure 3:**
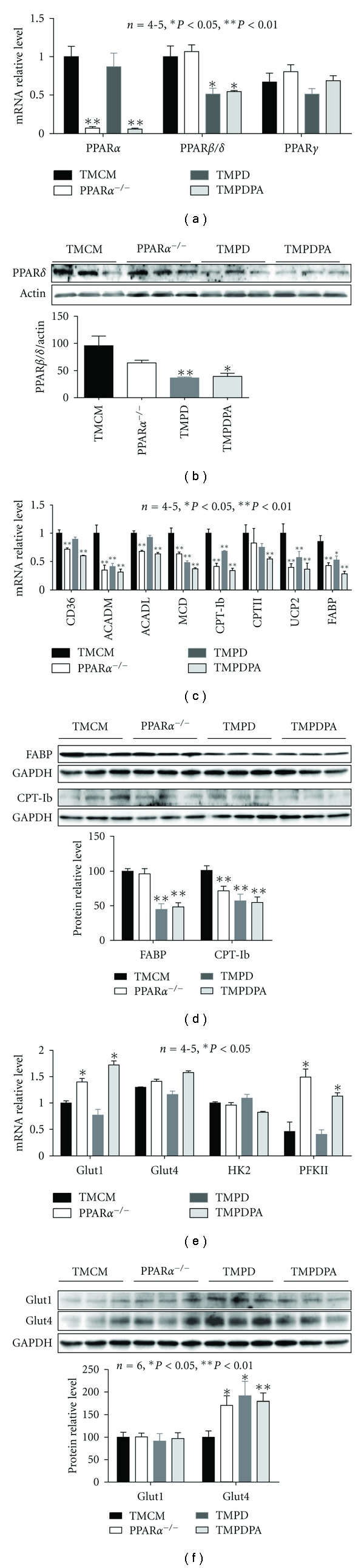
Expression of key proteins in fatty acid and glucose metabolism. (a) Real-time PCR results for transcript expression of PPAR*α*, PPAR*β*/*δ*, and PPAR*γ* on RNA samples extracted from ventricular tissues of TMCM, PPAR*α* ^−/−^, TMPD, and TMPDPA mice. (b) Western blotting results of PPAR*β*/*δ* protein levels in nuclear proteins from ventricular tissues of TMCM, PPAR*α* ^−/−^, TMPD, and TMPDPA mice. (c) Transcript level of fatty acid metabolism genes in samples from ventricular tissues of TMCM, PPAR*α* ^−/−^, TMPD, and TMPDPA mice. Expressions of CD36, ACADM, ACADL, MCD, CPT1b, CPTII, UCP2, and FABP are shown. (d) Protein levels of FABP and CPT-Ib in samples from ventricular tissues of TMCM, PPAR*α* ^−/−^, TMPD, and TMPDPA mice. (e) Transcript levels of Glut1, Glut4, HK2, and PFKII in samples from ventricular tissues of TMCM, PPAR*α* ^−/−^, TMPD, and TMPDPA mice. (f) Protein levels of Glut1 and Glut4 in samples from ventricular tissues of TMCM, PPAR*α* ^−/−^, TMPD, and TMPDPA mice. *P < 0.05  versus TMCM; **P < 0.01 versus TMCM.

To investigate the effects of short-term cardiac PPAR*β*/*δ* deficiency in adult PPAR*α*-null mice, we further studied tamoxifen inducible cardiomyocyte-restricted PPAR*β*/*δ*/PPAR*α* double knockout (TMPDPA) mice. As expected, PPAR*β*/*δ* was comparably decreased in the tamoxifen inducible cardiomyocyte-restricted PPAR*β*/*δ* knockout (TMPD) and TMPDPA hearts two weeks after tamoxifen injection, whereas it was unchanged in PPAR*α*-null and tamoxifen inducible MerCreMer (TMCM) hearts (Figure 3(a)). PPAR*γ* expression was not changed in any of the above mouse lines (Figure 3(a)). The TMPD and TMPDPA hearts showed decreased PPAR*β*/*δ* protein compared with PPAR*α*-null and TMCM hearts (Figure 3(b)). Consequently, transcript levels of representative genes encoding essential proteins in lipid metabolism, such as CD36, ACADM, acyl-Coenzyme A dehydrogenase long chain (ACADL), MCD, CPT-Ib, uncoupling protein 2 (UCP2), and FABP, were downregulated in PPAR*α*-null and TMPDPA compared with the control (TMCM) hearts (Figure 3(c)). Except for CD36 and ACADL, the above genes were also downregulated in TMPD hearts (Figure 3(c)). CPTII was decreased in PPAR*β*/*δ*-deficient hearts (Figure 3(c)). For protein levels, PPAR*α*-null hearts exhibited decreased CPT-Ib but not FABP (Figure 3(d)), whereas TMPD and TMPDPA hearts exhibited decreased CPT-Ib and FABP (Figure 3(d)). Transcript expression of proteins that are important in glucose metabolism, such as Glut1 and PFK, was markedly increased in mice with PPAR*α* deficiency (Figure 3(e)). Interestingly, Western blot revealed that Glut4, but not Glut1, was elevated in all the knockout (KO) lines (Figure 3(f)).

### 3.3. Short-Term Cardiac PPAR*β*/*δ* Deficiency Leads to Depressed Mitochondrial Biogenesis Independent of PPAR*α*

**Figure 4:**
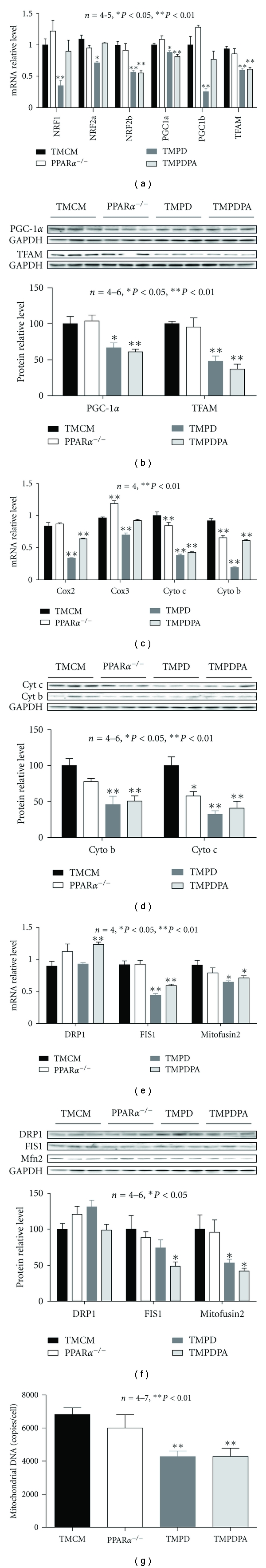
Expression of key determinants of mitochondrial biogenesis and mitochondrial proteins. (a) Real-time PCR measurement of transcript levels of NRF-1, NRF2 (a and b subunits), PGC-1*α* and -1*β*, and TFAM on samples from TMCM, PPAR*α* ^−/−^, TMPD, and TMPDPA hearts. (b) Western blotting analyses of relative protein levels of PGC-1*α* and TFAM on samples of nuclear proteins extracted from ventricular tissues of TMCM, PPAR*α* ^−/−^, TMPD, and TMPDPA mice. (c) Transcript expression of mitochondrial proteins on samples from TMCM, PPAR*α* ^−/−^, TMPD, and TMPDPA hearts. (d) Protein expression of mitochondrial proteins on samples from TMCM, PPAR*α* ^−/−^, TMPD, and TMPDPA hearts. (e) Transcript expression of mitochondrial fission and fusion proteins on samples from TMCM, PPAR*α* ^−/|−^, TMPD, and TMPDPA hearts. (f) Protein expression of mitochondrial fission and fusion proteins on samples from TMCM, PPAR*α* ^−/−^, TMPD, and TMPDPA hearts. (g) The mitochondrial DNA copy number on samples from TMCM, PPAR*α* ^−/−^, TMPD, and TMPDPA hearts. *P < 0.05 versus TMCM; **P < 0.01 versus TMCM.

**Figure 5:**
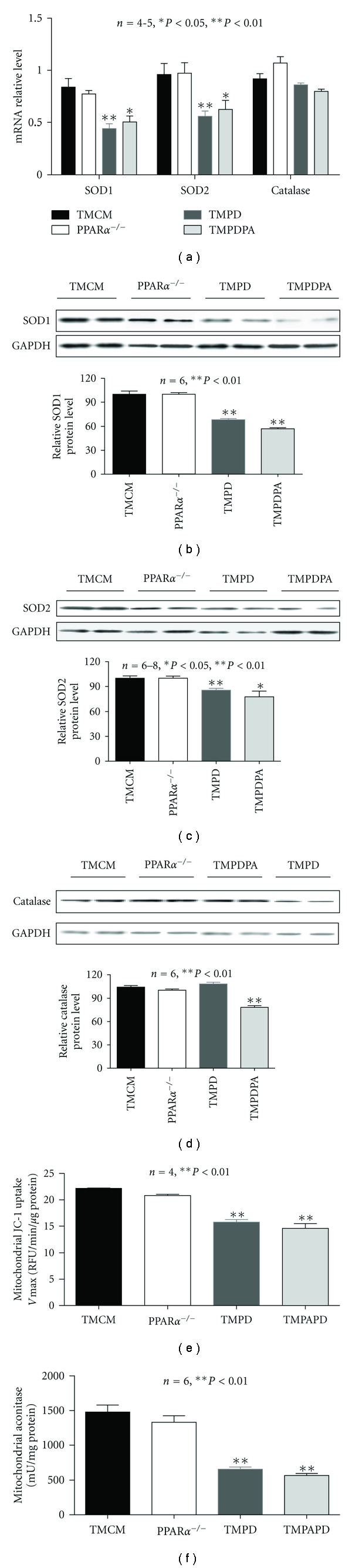
Endogenous anti oxidants and oxidative stress. (a) Real-time PCR analysis of transcript expression of SOD1, SOD2, and catalase on samples from TMCM, PPAR*α* ^−/−^, TMPD, and TMPDPA hearts. (b, c, and d) Western blotting analysis of protein levels of SOD1, SOD2, and catalase on samples from TMCM, PPAR*α* ^−/−^, TMPD, and TMPDPA hearts. (e) Mitochondrial membrane potential estimated by JC-1 uptake assay in isolated mitochondria from TMCM, PPAR*α* ^−/−^, TMPD, and TMPDPA hearts. (f) Mitochondrial aconitase activity of samples from TMCM, PPAR*α* ^−/−^, TMPD, and TMPDPA hearts. *P < 0.05 versus TMCM; **P < 0.01 versus TMCM.

Our recent study demonstrated that PPAR*β*/*δ* plays an essential role in regulating the transcriptional expression of key determinants of mitochondrial biogenesis [22]. Here we further assessed whether short-term additional PPAR*β*/*δ* deficiency in PPAR*α*-null mice would exacerbate this effect. Real-time PCR revealed that NRF1, NRF2a and b, and PGC1*α* and *β* were all downregulated in TMPD hearts (Figure 4(a)). In addition, mitochondrial transcription factor A (TFAM) was also downregulated in TMPD hearts (Figure 4(a)). In contrast with TMPD hearts, PPAR*α*-null hearts did not show changes in any of these important determinants of mitochondrial biogenesis (Figure 4(a)). The doubly deficient hearts displayed a comparable downregulation of the above genes, except for NRF1, NRF2a, and PGC1b (Figure 4(a)). Protein expression of key mitochondrial determinants, such as PGC-1*α* and TFAM, was decreased by about 30–40% and 50–60%, respectively, in TMPD and TMPDPA, but not in PPAR*α*-null hearts (Figure 4(b)). The transcript levels of mitochondrial proteins, such as cox2, cox3, cyto c, and cyto b, were downregulated in TMPD hearts, whereas cox2 and cyto c were downregulated in TMPDPA hearts (Figure 4(c)). There was no change in these transcript levels for PPAR*α*-null hearts (Figure 4(c)). Western blotting revealed that the protein levels of cyto b were downregulated in TMPD and TMPDPA hearts (Figure 4(d)). Interestingly, cardiac expression of cyto c protein was downregulated in all three PPAR-deficient lines (Figure 5(d)). The transcript expression of mitochondrial proteins involved in mitochondrial fission and fusion, such as Fis1 and mitofusin 2, was downregulated in both TMPD and TMPDPA hearts (Figure 4(e)). DRP1 was slightly increased in TMPDPA, but not in PPAR*α*- and PPAR*β*/*δ*-deficient hearts (Figure 4(e)). Protein levels of Fis1 were decreased in TMPDPA hearts (Figure 4(f)). Mitofusin 2 protein was decreased in both TMPD and TMPDPA, but not in PPAR*α*-null hearts (Figure 4(f)). Consequently, both TMPD and TMPDPA, but not PPAR*α*-null hearts, exhibited a similar attenuation of the mitochondrial DNA copy number (Figure 4(g)).

### 3.4. Short-Term Cardiac PPAR*β*/*δ* Deficiency Leads to Depressed Endogenous Anti Oxidants and Increased Oxidative Stress Independent of PPAR*α*

We next examined whether PPAR*α* is involved in the transcriptional regulation of important endogenous anti oxidants in the heart. The transcript and protein expression of both SOD1 and SOD2 was decreased in TMPD and TMPDPA, but not in PPAR*α*-null hearts, relative to control hearts (TMCM) (Figure 5(a)). Protein expression of both SOD1 and SOD2 was decreased in TMPD and TMPDPA hearts (Figures 5(b) and 5(c)). Interestingly, catalase protein expression was decreased only in TMPDPA hearts (Figure 5(d)). TMPD and TMPDPA, but not PPAR*α*-null, hearts exhibited augmented oxidative stress, illustrated by depressed aconitase activity and mitochondrial membrane potential (JC-1) compared with controls (Figures 5(e) and 5(f)).

### 3.5. Short-Term Cardiac PPAR*β*/*δ* Deficiency Leads to Depressed Myocardial Fatty Acid Oxidation, Cardiac Dysfunction, and Cardiac Hypertrophy Independent of PPAR*α*

**Figure 6:**
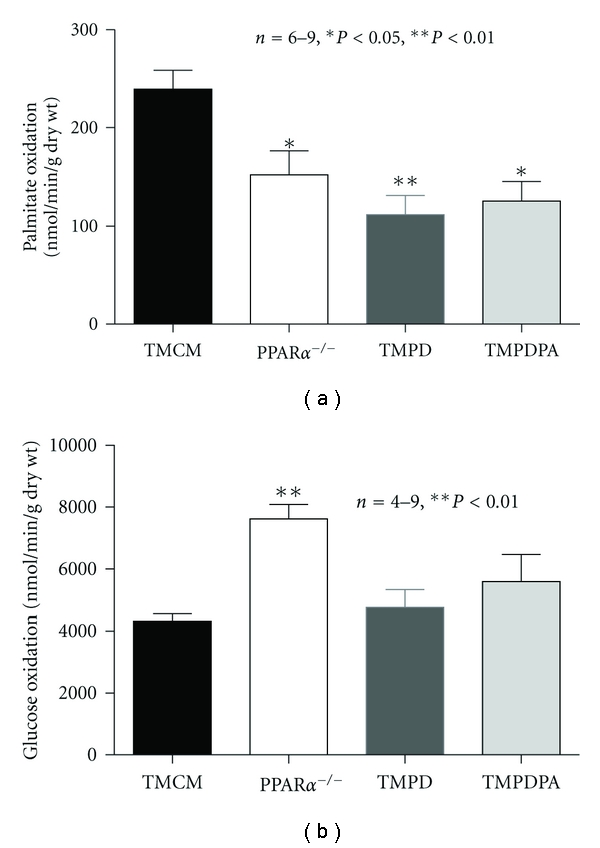
Rates of oxidative metabolism and cardiac function. (a) The rate of palmitate oxidation in isolated working hearts from TMCM, PPAR*α* ^−/−^, TMPD, and TMPDPA mice was measured using ^2^H_2_O-labeled palmitate. (b) The rate of glucose oxidation in isolated working hearts from TMCM, PPAR*α* ^−/−^, TMPD, and TMPDPA mice was measured using ^14^C-labeled glucose. *P < 0.05 versus TMCM; **P < 0.01 versus TMCM.

We assessed the rate of myocardial oxidative metabolism and cardiac function in the isolated working heart for the four experimental groups. As expected, the palmitate oxidation rates in PPAR*α*-null, TMPD, and TMPDPA hearts were similarly downregulated compared with control mice (TMCM) (Figure 6(a)). Only PPAR*α*-null hearts showed an increase in glucose oxidation (Figure 6(b)). The isolated working heart studies revealed that short-term deficiency of PPAR*β*/*δ* led to depression of cardiac contraction with decreased left-ventricular systolic pressure (LVSP), LV developed pressure (LVPamp), and dLVPd*t*_max_ (Table 1). Noninvasive echocardiographic measurement revealed that TMPD and TMPDPA hearts displayed increased intraleft ventricular dimension (LVID) with thickening intraventricular septa (IVS), increased LV mass, and decreased ejection fraction (Table 2). Fractional shortening and the rate of deceleration of mitral valve flow (MV Decel rate) were decreased (Table 2). 

## 4. Discussion

The coexistence of the three PPAR subtypes in cardiomyocytes is now well established [4, 6, 10–12, 29–31]. It becomes clear that the three PPAR subtypes share many of their functions in the transcriptional regulation of lipid metabolism [10, 11, 26, 29]. This is especially true for PPAR*α* and PPAR*β*/*δ*, which are relatively abundant in cardiomyocytes. However, there are still gaps in our knowledge regarding the interrelationship of these subtypes. In the present study, we test the hypothesis that PPAR*α* is required for the detrimental effects of PPAR*β*/*δ* deficiency in the heart on myocardial FAO and mitochondrial biology. It is a surprise that cardiomyocyte-restricted deletion of PPAR*β*/*δ* in PPAR*α*-null mice causes impaired mitochondrial biogenesis and defense, but no further depression of FAO. 

The most striking pathological changes shared by PPAR*β*/*δ* KO and PPAR*α*/PPAR*β*/*δ* double KO hearts are mitochondrial abnormalities, such as shrinkage and depletion. Mitochondrial DNA copy numbers were also substantially decreased. There were many lipid droplets with large sizes and disrupted sarcomere found in their ultrastructure. To minimize potential confounding effects of development (before adulthood) and severe pathology in long-term PPAR*β*/*δ*-deficient hearts, we further investigated mice with short-term (~14 days after tamoxifen induction) cardiomyocyte-restricted PPAR*β*/*δ* (TMPD) in the adult heart. As expected, the transcriptional expression of key determinants of mitochondrial biogenesis, along with many mitochondrial proteins, was decreased in PPAR*β*/*δ*-deficient hearts with or without PPAR*α* compared with PPAR*α*-null and controlled hearts. The expression of anti oxidants, such as SOD1 and SOD2, was also the same. As a result, mitochondrial DNA copy number and mitochondrial oxidative stress were impaired only in TMPD and TMPDPA mouse hearts. Therefore, these deficiencies should be attributed to the phenotypic changes in mice with PPAR*β*/*δ* deficiency, supporting the notion that cardiac PPAR*β*/*δ* is an independent, essential transcriptional regulator of mitochondrial biogenesis and the mitochondrial anti-oxidant defense system in the heart. Cardiac PPAR*α* may not be required or its deficiency may be compensated by unknown mechanisms. It has been shown that PPAR*α* determines mitochondrial biogenic responses in insulin-resistant hearts [8]. Therefore, it is possible that the effects of PPAR*α* on myocardial mitochondrial biogenesis are responsive only to specific physiological or pathological stresses, which is similar to its response to fasting condition [7, 18]. 

It is surprising that cardiac deficiency induced by PPAR*α*/PPAR*β*/*δ* deficiencies did not further impair the expression of most FAO genes. It is plausible that the heart possesses other mechanisms for maintaining a minimal level of lipid metabolism. We recently showed that PPAR*γ* in the adult heart also plays a role in regulating fatty acid utilization [32]. Although PPAR*γ* expression was not changed in any of the experimental groups in this study, we could not rule out the possibility that PPAR*γ* helps maintain the minimal level of oxidative metabolism via increasing its activity. Although the PPAR*α*-null heart displayed a similarly suppressed myocardial lipid metabolism, no overt phenotype could be detected in these mice under basal conditions during adulthood. In contrast, phenotypic changes in the long-term double KO mice were largely identical to those of PPAR*β*/*δ* monogenic KO mice [12] with the progressive development of cardiac hypertrophy, mitochondrial depletion, and premature death. Therefore, it appears that impaired mitochondrial biogenesis and exacerbated oxidative damages, in addition to myocardial FAO deficiency, accounts, for the severe phenotypic changes in PPAR*β*/*δ*-deficient hearts. 

Even though cardiac PPAR*β*/*δ* KO in TMPD mice appears to be less complete compared with CR-PPAR*β*/*δ* ^−/−^ mice, it is sufficient to impair the expression of major lipid metabolism genes. In fact, the PPAR*α* and PPAR*β*/*δ* single KO and the double KO lines exhibited similarly impaired rates of myocardial FAO. While myocardial lipid accumulation is consistently increased in PPAR*β*/*δ*-deficient mouse hearts with or without PPAR*α*, this is not as apparent in the short-term PPAR*β*/*δ*-deficient hearts. It is therefore likely, but remains to be proven, that the adult heart possesses a greater capacity for handling excessive unburned fat than does the newborn heart. However, the void of PPAR*β*/*δ* in the adult heart is sufficient to cause cardiac dysfunction. *Ex vivo *and *in vivo* cardiac function assessment revealed that both systolic and diastolic dysfunction can be detected in mice with PPAR*β*/*δ* deficiency in their hearts, consistent with the increased left ventricular mass. 

Interestingly, expression of Glut1 and PFKII transcripts was increased in both PPAR*α*-null and the TMPDPA, but not in PPAR*β*/*δ* single deficient hearts. Moreover, Glut4 protein levels were upregulated in all PPAR deficient hearts, but only PPAR*α*-null hearts exhibited greater rates of glucose oxidation. TMPD hearts maintained normal glucose oxidation in this set of study and was decreased in the previous study [22]. The lack of dysfunction in the PPAR*α*-null compared with PPAR*β*/*δ* deficiency hearts could be either of the following (1) normal mitochondria allowing greater capacity in glucose oxidation and insulin sensitivity: and (2) differences in the storage or types of stored lipids. It is likely that the impaired mitochondrial capacity due to PPAR*β*/*δ* deficiency prevents the upregulation of glucose oxidation. The upregulation of glucose utilization should be attributed to the well-maintained cardiac function in PPAR*α*-null hearts. It is conceivable that the compensatory responses occur at both transcriptional and translational levels. However, it is obvious that this compensation is not sufficient for the TMPDPA heart to overcome the mitochondrial defects derived from PPAR*β*/*δ* deficiency. It is intriguing that PPAR*β*/*δ* deficiency in cultured adult cardiomyocytes leads to depression of both FAO and glucose oxidation [22]. However, the current study based on isolated hearts revealed that FAO, but not glucose oxidation, was depressed in PPAR*β*/*δ*-deficient hearts. It is likely that glucose oxidation is better conserved in the *ex vivo* hearts or it is due to indirect compensatory upregulation similar to, but not as robust as, those in PPAR*α*-null hearts. Indirect responses may also be the causes of certain disagreements in the expression pattern between the transcript and protein levels. For example, cardiac expression of FABP was downregulated at transcript level but not at protein level in PPAR*α*-null hearts. Slight differences in time points, sample handling, tamoxifen absorption, reagents from different lots may also contribute to the inconsistencies. However, the overall pathophysiological changes are highly consistent in the short-term PPAR*β*/*δ*-deficient hearts in this study and the previous study [22]. 

Although double knockout of PPAR*α* and PPAR*β*/*δ* led to largely similar depression in expression of key metabolic and mitochondrial proteins, it is noted that certain degrees of interaction between the two PPAR subtypes may occur in the double knockout heart. Compared with the control and TMPD hearts, Glut1 and PFKII transcripts were upregulated; NRF1, NRF2a, and PCG1b transcripts were unchanged in double deficient hearts. However, these potential changes due to the voids of both PPAR subtypes appear to be insufficient to rescue the impaired mitochondrial structure/function. Burkart et al. have previously demonstrated in gain-of-function studies that PPAR*α* and PPAR*β*/*δ* have distinct roles in regulating glucose metabolism. Specifically, they documented differential regulation of Glut4 via a myocyte enhancer factor (MEF) response element [13]. It is plausible that an upregulation of cardiac Glut4 found in the three PPAR-deficient lines is due to compensatory responses regulating by MEF pathway and/or other signaling pathways. 

In summary, the present study demonstrates that the double deficiency of PPAR*α* and PPAR*β*/*δ* in the heart does not aggravate the impairment of myocardial FAO. PPAR*α* deficiency does not alter the unique function of PPAR*β*/*δ* as an essential regulator of cardiac mitochondrial protection and biogenesis in the heart. Therefore, we conclude that PPAR*β*/*δ* is essential for myocardial mitochondrial function independent of PPAR*α*.

##  Authors' Contribution

J. Liu and P. Wang contributed equally to this work.

## Supplementary Material

Supplemental file 1: A table contains information of primers used in Real Time PCR experiments to detect transcript expression levels.
Supplemental File 2: Supplemental figures demonstrate changes in mitochondrial volume and DNA copy number: The mitochondrial volume was quantified based on electron micrographs of *α*-MyHC-Cre, PPAR*α*-/- , CR-PPAR*β*/*δ* -/- , CR-Pd/Pa null heart sections. Mitochondrial DNA copy number was measured by Real time PCR.

## Figures and Tables

**Table 1:** Hemodynamic measurement of isolated working heart.

Parameters	TMCM	Pa	TMPD	TMPDPA
HR	431 ± 20	435 ± 22	410 ± 23	406 ± 16
LVPsys (mmHg)	110.8 ± 3.9	110.5 ± 2.3	94.7 ± 5.6*	105.0 ± 1.7
LVEDP (mmHg)	7.8 ± 1.8	7.8 ± 1.6	9.8 ± 2	12.5 ± 2.8
LVPdia (mmHg)	2.4 ± 1.3	3.7 ± 1.2	5.7 ± 1.8	7.3 ± 3.4
LVPamp (mmHg)	108.4 ± 3.8	106.8 ± 2.6	84.0 ± 5.7**	91.7 ± 5.3*
dLVP d*t*_max_ (mmHg/s)	6691 ± 518	5999.7 ± 361	5154 ± 332*	5500.9 ± 683.7
−dLVP d*t*_min_ (mmHg/s)	−5666 ± 428	−4741 ± 330.6	−4635.8 ± 338	−4836.2 ± 614.9

Heart rate: HR; left ventricular systolic pressure: LVPsys; left ventricular end-diastolic pressure: LVEDP; left ventricular diastolic pressure: LVPdia; left ventricular developed pressure: LVPamp; left ventricular developed pressure; first derivative (dLVPd*t*_max_ and dLVPd*t*_min_), *P < 0.05 versus TMCM, *n* = 5–8.

**Table 2:** Echocardiography measurement in mice ~two weeks after the end of tamoxifen treatment.

Parameters	TMCM	Pa	TMPD	TMPDPA
IVS;d (mm)	0.77 ± 0.02	0.81 ± 0.04	0.95 ± 0.06*	0.96 ± 0.02*
IVS;s (mm)	1.29 ± 0.06	1.16 ± 0.08	1.30 ± 0.05	1.12 ± 0.05
LVID;d (mm)	3.65 ± 0.06	3.85 ± 0.11	3.91 ± 0.11	3.65 ± 0.09
LVID;s (mm)	2.2 ± 0.09	2.12 ± 0.09	2.54 ± 0.11*	2.62 ± 0.09*
LVPW;d (mm)	0.69 ± 0.03	0.67 ± 0.05	0.76 ± 0.04	0.61 ± 0.02
LVPW;s (mm)	1.13 ± 0.04	0.99 ± 0.06	1.13 ± 0.06	1.05 ± 0.02
Ejection Fraction (%)	71.5 ± 1.5	73.66 ± 1.68	64.71 ± 2.41*	60.57 ± 2.31*
Fractional Shortening (%)	40.1 ± 2	41.9 ± 1.39	35.01 ± 1.84*	31.93 ± 1.59*
MV Decel rate (mm/s^2^)	−38144 ± 1666	−30433 ± 2693	−23192 ± 828*	−19992 ± 2873*
HR (Beat/Min)	414 ± 7	406 ± 13	417 ± 25	390 ± 14
LV mass/BW	3.29 ± 0.13	3.50 ± 0.1	4.05 ± 0.2*	4.05 ± 0.12*

IVS;d and IVS;s: interventricular septum (diastole and systole); LVID;d and LVID;s: left ventricular internal diameter (diastole and systole); LVPW;d and LVPW;s: left ventricular posterior wall (diastole and systole); EF%: ejection fraction; FS%: fractional shortening. MV decal rate: mitral valve deceleration rate; HR: heart rate, LV mass/BW: ratio of left ventricular mass to body weight. *P < 0.05 versus TMCM *n* = 8.

## Abbreviations

ACADL: acyl-Coenzyme A dehydrogenase long chain
ACADM: acyl-Coenzyme A dehydrogenase medium chain
Cox: Cytochrome c oxidase
CPT-Ib: carnitine palmitoyltransferase-Ib
Cyto b: Cytochrome b5
Cyto c: cytochrome c
DRP1: mitochondrial fission 1
FATP: fatty acid transport protein
FABP: fatty acid binding protein, heart type
FAO: fatty acid oxidation
Fis1: mitochondrial fission 1 protein
MCD: Malonyl-CoA decarboxylase
Mfn: mitofusin
NRF: nuclear respiratory factor
PGC-1: PPAR*γ* co-activator 1
rcan1: regulator of calcineurin 1
TEM: transmission electron microscopy
TFAM: mitochondrial transcription factor A
TMCM: tamoxifen inducible MerCreMer
UCP2: uncoupling protein 2
