# Supplementary material for: Cardiomyocyte-Restricted Deletion of PPARβ/δ in PPARα-Null Mice Causes Impaired Mitochondrial Biogenesis and Defense, but No Further Depression of Myocardial Fatty Acid Oxidation
Source: PPAR Res. 2011 Sep 5;2011:372854. doi: 10.1155/2011/372854 (PMC3167180; doi:10.1155/2011/372854)
Supplement: Supplementary file 1 — Supplemental file 1: A table contains information of primers used in Real Time PCR experiments to detect transcript expression levels. Supplemental File 2: Supplemental figures demonstrate changes in mitochondrial volume and DNA copy number: The mitochondrial volume was quantified based on electron micrographs of α-MyHC-Cre, PPARα-/- , CR-PPARβ/δ -/- , CR-Pd/Pa null heart sections. Mitochondrial DNA copy number was measured by Real time PCR. [file 372854.f1.pdf]

Supplemental Table 1: Primers used in the present study

Gene	Forward	Reverse
b-actin	5'CTGTCCCTGTATGCCTCTG 3'	5'ATGTCACGCACGATTTCC 3'
FABP-h	5'TCGAGAAGAACGGGGATAC 3'	5'CTGCCATGGGTGAGAGT 3'
CD36	5'AAAGTTGCCATAATTGAGTCCT 3'	5'TCCGAACACAGCGTAGATAGA 3'
ACS	5'GCAAGAAGTGTGGGGTGGAAATCA 3'	5'CATATGGGCGAGAGGCAAGAAAGA 3'
Acadm	5'ACTCGCTAGGCTCAGTTACCA 3'	5'GTCCCTCATCAGCTTCTCCACA 3'
Acadl	5'CCGCCCGATGTTCTCATTCTG 3'	5'TCTGCGATGTTGATGCCAAGC 3'
MCD	5'ATGAGGCTGTGTGCCTGGTA 3'	5'TTGCTGTTGTTCTGGAACTGG 3'
Cpt1b	5'TTCAACACTACACGCATCCC 3'	5'GCCCTCATAGAGCCAGACC 3'
Cpt II	5'GCTCCGAGGCATTTGTC 3'	5' CATCGCTGCTTCTTTGGT 3'
UCP2	5'TTGGTTTCAAGGCCACAGAT 3'	5'TACCCAGAACGCCACGGTA 3'
GPAT	5'GGAGTGTGGCGAGAGGCGTTATC 3'	5'TTGCTGGCGGTGAAGAGAATGTG 3'
PFK	5'GCCGTGTTGACCTCTGG 3'	5'TCGTCCTTCTCGCTCCC 3'
HK2	5'TTTTAGGTCAGTCGGCGTTTC 3'	5'CTCGATGCAGCTCAGTCCAAG 3'
Glut1	5'GAAGAGGGTCGGCAGATGA 3'	5'CGAAGATGCTCGTTGAGTAGTAGA 3'
Glut4	5'TTCCAGCAGATCGGCTCTGA 3'	5'AAGACATTGTTGGCCAGCAT 3'
PPARa	5' ATTTCCCTCTTTGTGGCTGCTAT 3'	5' GCGTCGGACTCGGTCTTCTTGAT 3'
PPARd	5'TCGGGCTTCCACTACGG 3'	5'ACTGACACTTGTTGCGGTTCT 3'
PPARg	5'GCCATTGAGTGCCGAGTCTGT 3'	5'GCATCCGCCCAAACCTGA 3'
SOD1	5'GGAAGCATGGCGATGAAA 3'	5'AAATGAGGTCCTGCACTGGTA 3'
SOD2	5'GCCTCCCAGACCTGCCTTAC 3'	5'TCGGTGGCGTTGAGATTGT 3'
catalase	5'AAGGTTTGGCCTCACAAGGA 3'	5'GCGGTAGGGACAGTTCACAG 3'
TFAM	5'AGCAGCAGGCACTACAGCGATAC 3'	5'TTCCCATTCCCTTCCCAGAC 3'
PGC1a	5'TGTTCCCGATCACCATATTCC 3'	5'TCCCGCTTCTCGTGCTCTTT 3'
PGC1b	5'AGGTGTTCGGTGAGATTGTA 3'	5'TCAGATGTGGGATCATAGTCA 3'
NRF1	5'CATGGAGGAGCACGGAGTGA 3'	5'AGCAGCCAGATGGGCAGTTA 3'
NRF2a	5'ACGGCACCAAGCACATTACGAC 3'	5'GAACTCCTTCATTACCCAAACC 3'
NRF2b	5'GTGTGGGGGGCGGGGTAT 3'	5'CCTGGCTCTGAGGGGTTT 3'
Mfn2	5'AAGTCCGGGAAGCTGAAAGT 3'	5'TCTCGGTTATGGAACCAACC 3'
DRP1	5'CAGCCCAAGGACATCG 3'	5'TGGCATCAGTACCCGC 3'
FIS1	5'CTGAGCCCCAGAACAACCA 3'	5'CCCTCTTCCAGTGTCCCCC 3'
Cox2	5'ACCTGGTGAACTACGACTGCTA 3'	5'GTCGGTTTGATGTTACTGTTGC 3'
Cox3	5'GCAGGATTCTTCTGAGCGTTCTA 3'	5' AGGGCTTGATTTATGTGGTTTCGT 3'
Cytob	5'ATGACAGATCAAAGATAGCCAAGC 3'	5'GCACAGGAGGAAAGGGAAAC 3'
Cyto c	5'GCCTCTATTTCAACCCTTACTTT 3'	5'TGACCACTTATGCCGCTTC 3'
Thiolase	5'AAGACCTGATTGTAAAAGACGG 3'	5' CACTGAGATGGTGATGGGAG 3'
