# Supplementary material for: Cardiomyocyte-Restricted Deletion of PPARβ/δ in PPARα-Null Mice Causes Impaired Mitochondrial Biogenesis and Defense, but No Further Depression of Myocardial Fatty Acid Oxidation
Source: PPAR Res. 2011 Sep 5;2011:372854. doi: 10.1155/2011/372854 (PMC3167180; doi:10.1155/2011/372854)
Supplement: Supplementary file 2 [file 372854.f2.pdf]

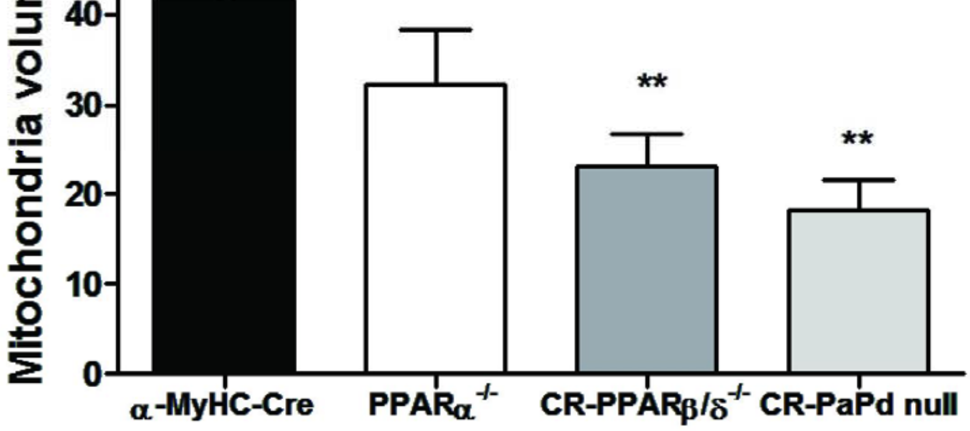

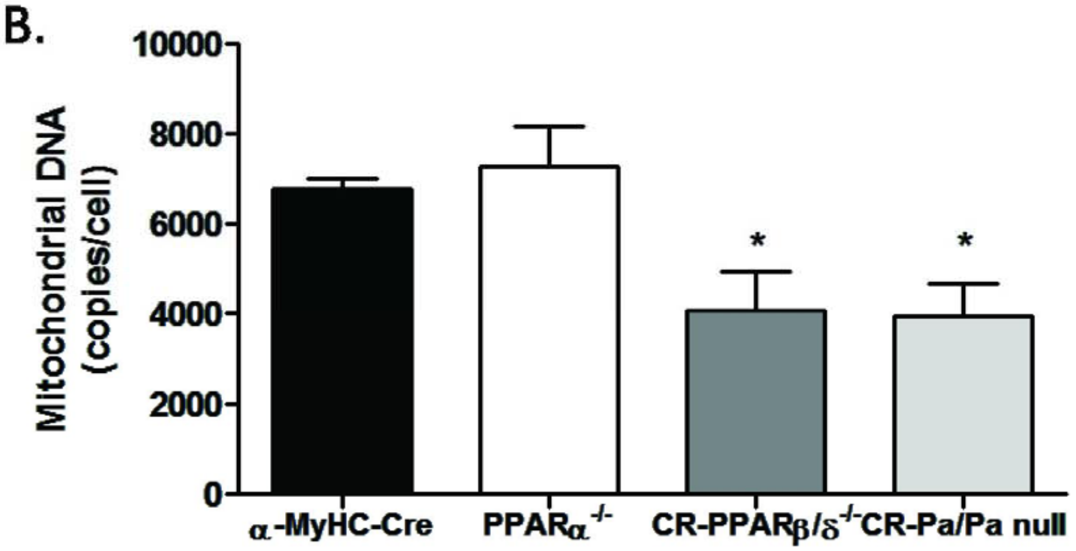

Sup. Figure 1 Mitochondrial volume and DNA copy number A) Volume of mitochondria analyzed on electron micrographs (x12000) of α-MyHC-Cre, PPARα$^{-/-}$, CR-PPARβ/δ $^{-/-}$, CR-Pd/Pa null heart sections. Samples are from ventricular tissues of 4-month-old mice. The volume of mitochondria was expressed as a percentage of the total area in the TEM micrographs. Data are from 4-7 randomly selected images. **B)** The Mitochondrial DNA copy number on samples from α-MyHC-Cre, PPARα$^{-/-}$, CR-PPARβ/δ $^{-/-}$, CR-Pd/Pa null hearts. **p<0.05 vs* α-MyHC-Cre, and ***p<0.01 vs* α-MyHC-Cre, n=6.
